# Measurement of the Photothermal Conversion Efficiency of CNT Films Utilizing a Raman Spectrum

**DOI:** 10.3390/nano12071101

**Published:** 2022-03-27

**Authors:** Yu Liu, Zhicheng Lin, Pengfei Wang, Feng Huang, Jia-Lin Sun

**Affiliations:** 1 College of Mechanical Engineering and Automation, Fuzhou University, Fuzhou 350108, China; liuyu19@fzu.edu.cn (Y.L.); 15160454010@163.com (Z.L.); wangpf@fzu.edu.cn (P.W.); huangf@fzu.edu.cn (F.H.); 2 State Key Laboratory of Low-Dimensional Quantum Physics, Department of Physics, Tsinghua University, Beijing 100084, China

**Keywords:** CNT film, Raman shift, photothermal conversion efficiency

## Abstract

Because carbon nanotube (CNT) films have high photothermal conversion efficiency (PTCE), they have been widely used in bolometric and photothermoelectric photodetectors, seawater desalination, and cancer therapy. Here, we present a simple, quick, and non-destructive method to measure the PTCE of CNT films. According to the linear relationship between the Raman shift of the G^+^ peak and the temperature of a CNT, the offset of the G^+^ peak under varying excitation light power can characterize the changed temperature. Combining the simulation of the temperature distribution, the final value of the PTCE can be obtained. Finally, a CNT film with a high PTCE was chosen to be fabricated as a bolometric photodetector; a quite high responsivity (2 A W^−1^ at 532 nm) of this device demonstrated the effectiveness of our method.

## 1. Introduction

Carbon nanotubes (CNTs) are widely used in seawater desalination [1,2,3,4,5,6], environmental protection [7], thermal energy storage [8,9,10,11], photothermal dynamic therapy [12,13,14], and photodetectors [15,16,17,18] because of their efficient photothermal conversion ability. However, the synthesis process of CNTs is difficult to control strictly, which often induces complex morphologies and chirality distribution; simultaneously, some defects and impurities are also introduced. These factors will lead to significant differences between the values of the photothermal conversion efficiency (PTCE) of CNTs. Therefore, the measurement of the PTCE before practical applications is a critical step that can effectively help to identify the CNT materials with a high PTCE.

Previously, the general methods for measuring PTCE used infrared cameras or infrared photodetectors to observe the temperature changes. These methods always exhibited some drawbacks of low accuracy, small dynamic range, poor resolution, and great damage [19,20,21]. Here, we proposed a simple, quick, and non-destructive method to measure the PTCE of CNT films based on a Raman spectrum. Due to the linear relationship between the Raman shift of the G^+^ peak and the temperature of a CNT [22,23,24], the changed temperature of a CNT film under laser excitation can be equivalently expressed as the offset of the G^+^ peak. The value of the PTCE was calculated through integrating the simulated temperature distribution. The results indicated that the impurities and defects can significantly reduce the PTCE of CNT films and fibers. The PTCE of a pure CNT film was 4.8 times larger than that of a dirty one. In another sample, the PTCE for a position with few defects was 2.5 times larger than that for a position with many defects. This method was able to very quickly compare the PCTE values among different CNT materials, and to identify ones with high PTCE. Finally, a CNT film with the highest PTCE was used to fabricate a bolometric photodetector. The responsivity of the device under illumination of 532 nm laser reached 2 A W^−1^, which was the highest among the bolometric photodetectors based on CNTs [15,16,25].

## 2. Materials and Methods

The CNT films used in this paper were synthesized following the CVD method in a previous report, and the process of fabricating a suspended structure also followed that work [26]. The CNT fibers were drawn from an as-prepared CNT film. Then, the CNT film or fibers were stretched between two substrates. Alcohol was used to wet the CNT materials to promote intimate contact between the CNT materials and the substrates (deposited Au/Ti on SiO_2_) [15].

The relationship between the Raman shift of the G^+^ peak and the temperature of a CNT film was calibrated by a high-resolution Raman spectrometer (HORIBA T64000, Kyoto, Japan) with a tunable thermostatic sample cavity. Then, the Raman shift of the G^+^ peaks under different excitation powers were measured by a Laser Confocal Raman Microscope (WITec Alpha 300RAS, Stuttgart, Germany). A laser with a wavelength of 532 nm was used as an excitation light, which heated and also probed the samples. An objective (Zeiss EC Epiplan- Neofluar 100×/0.9, Oberkochen, Germany) was used to focus the laser beam to a spot with a diameter of 0.5 μm. The integration time was 0.7 s and the number of accumulations was 10. A SourceMeter (Keithley 2400, Beaverton, OR, USA) unit was used to characterize the photoresponse of the bolometric photodetector under the illumination of a 532 nm laser.

## 3. Results

**Figure 1:**
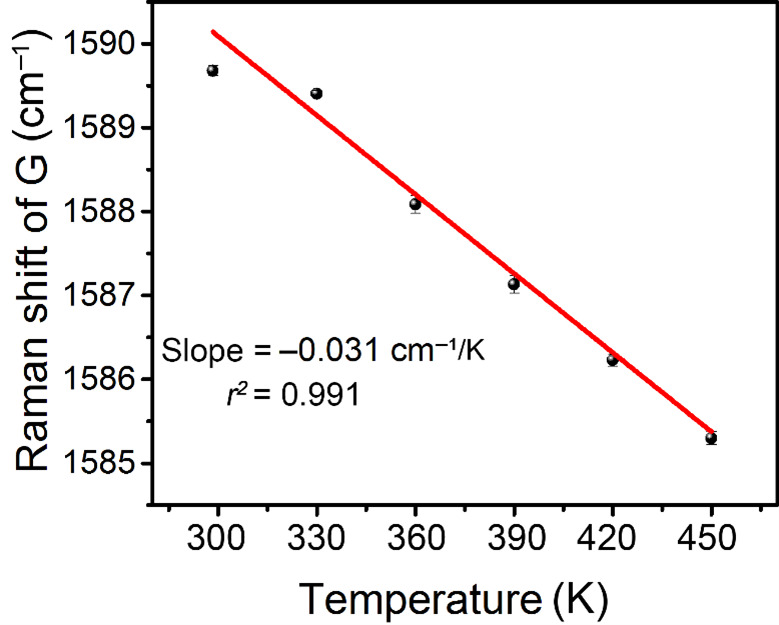
Raman shift of G^+^ peak as a function of the temperature for CNT films.

Several previous articles demonstrated that the temperature of a single CNT is linearly related to the Raman shift of the G^+^ peak [22,23,24]. Further, the temperature of a CNT film remained linearly related to the Raman shift of the G^+^ peak [27]. Here, we also measured the Raman shift of the G^+^ peak under different temperatures, as Figure 1 shows [28]. The Raman shift of the G^+^ peak exhibited a linear relationship with the temperature, with a slope of −0.031 cm^−1^ K^−1^, which was a little larger than the previously reported value [27]. This difference in slopes is possibly due to the suspended structure, because the stress within suspended CNT film is larger than that in a supported structure, and the stress can also change the Raman shift of the G^+^ peak [29].

**Figure 2:**
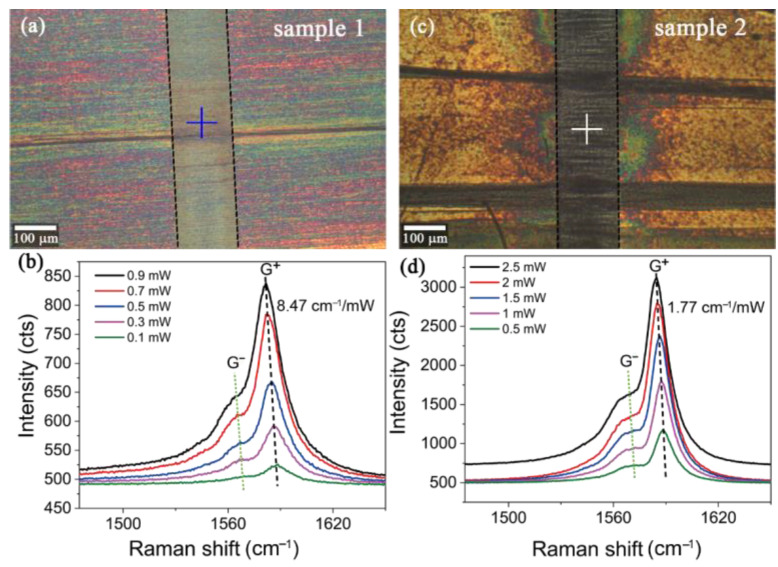
(**a**) Optical image of Sample 1; (**b**) Raman spectra of Sample 1 under different excitation powers; (**c**) Optical image of Sample 2; (**d**) Raman spectra of Sample 2 under different excitation powers. The middle part between the two black dash lines is the suspended CNT film. The widths of the trenches for Sample 1 and Sample 2 are both 120 μm. The crosses mark the probe points.

Figure 2 shows the optical images and the Raman spectral under different excitation light powers for Sample 1 and Sample 2. Figure 2b,d shows that both of the CNT films have the same experimental phenomenon, i.e., that a higher excitation light power induces a red shift of the G^+^ peak. If we define a proportionality coefficient *R* = Δω/Δ*p*, where Δω and Δ*p* represent the offset value of the G^+^ peak and the increased light power respectively, the *R* of Sample 1 (8.47 cm^−1^ mW^−1^) is almost five times larger than that of Sample 2 (1.77 cm^−1^ mW^−1^). Therefore, together with the result in Figure 1, the ratio of the increased temperature to the light power at the tested point in Sample 1 was obtained as 273 K mW^−1^ and the value in Sample 2 was 57 K mW^−1^. However, when the changed temperature was high enough, the relationship would be non-linear; this condition was not considered in this work.

**Figure 3:**
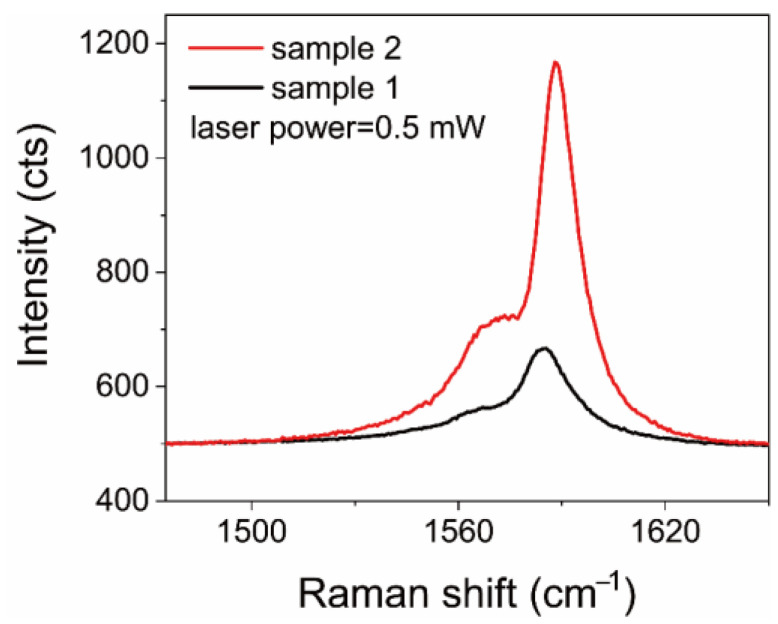
Raman spectra of Sample 1 and Sample 2.

The experimental results indicated that Sample 1 could better convert the incident photon energy into heat. Comparing the optical images of Sample 1 and Sample 2, it is obvious that the surface of Sample 1 is cleaner, but a large number of impurities (metal catalysts and amorphous carbon) can be observed on the surface of Sample 2 (see Figure S1a,c). In addition, Figure 3 exhibits that under the same excitation light power, the intensity of the G^+^ peak in Sample 2 is much larger than that in Sample 1, indicating that these impurities on the surface can greatly enhance the Raman scattering intensity. During the synthesis process of the CNT films, iron and copper nanoparticles were introduced as catalysts (see Figure S1b,d). Hence, the surface-enhanced Raman scattering from iron nanoparticles could dominate the enhanced intensity of the G^+^ peak for Sample 2 [30,31,32,33]. According to previous reports, the ratio of *ω*_G_^−^ (the Raman shift of the G^−^ peak) to the temperature is the same as with *ω*_G_^+^, which was also demonstrated in our work, as the green dash lines show in Figure 2b,d [22,23,24]. Together, these results indicate that impurities could dramatically weaken the ability of photothermal conversion for a CNT film.

**Figure 4:**
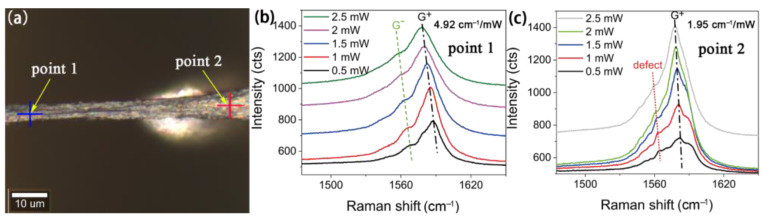
(**a**) Optical image of Sample 3, in which the position of blue cross marks point 1 and the position of red cross marks point 2; (**b**) Raman spectra of point 1 at different excitation light powers; (**c**) Raman spectra of point 2 at different excitation light powers.

The same experiments were also conducted on a CNT fiber noted as Sample 3, and the experimental results are shown in Figure 4. The *R* of point 1 in Sample 3 is greater than that of point 2. The Raman spectra of point 2 display some obvious shoulder peaks that show a linear relationship with the excitation light power, but the slope is different from the G^+^ peak. Hence, these shoulder peaks were possibly due to defects. Some reported works demonstrated that the existence of defects and impurities in carbon nanotubes had a greatly negative impact on photothermal conversion [34], which was very consistent with our experimental results. According to the optical image of Sample 3, it is obvious that point 2 is closer to the edge of the suspended film. Therefore, we assumed that the defects may be introduced by the strong tensile effect at the edge, or by damage during the transfer processes [35].

## 4. Discussion

The photothermal conversion efficiency *η* of CNT film can be expressed as:

(1) η=Q/P

where *Q* is the heat generating power of the CNT film (generated heat per unit time) and *P* is the incident light power [36].

Under the illumination of a continuing laser, a CNT film absorbs the light energy and converts it into heat. Some of the heat can transfer to the surrounding air, and the remaining heat will increase the temperature of the CNT film. Therefore, the stored heat within the CNT film per unit time can be expressed as:

(2) $$Q-Q_{surr}=c\times \int_{\Sigma }\rho \cdot h\cdot (dT(x,y)/dt)\cdot ds$$

where *Q_surr_* is the heat dissipation power from the CNT film to air, *c* is the specific heat capacity of the CNT film, *ρ* is the density of the CNT film, *h* is the thickness of the CNT film, *T*(*x*, *y*) is the temperature distribution of the CNT film, and *t* is time.

After a fast process of increasing temperature, the heat generated by the CNT film and the heat dissipating to air will become equal. Therefore, the temperature of the CNT film will remain stable. The heat-generating power at the thermal equilibrium state can be expressed as:

(3) $$Q=Q_{surr}=2G_{0}\times \int_{\Sigma }(T-T_{0})\cdot ds$$

where *G_0_* is the heat transfer coefficient of air and *T_0_* is the room temperature, 300 K. Therefore, the PTCE can be expressed as the ratio of *Q_surr_* to incident light power *P*.

**Figure 5:**
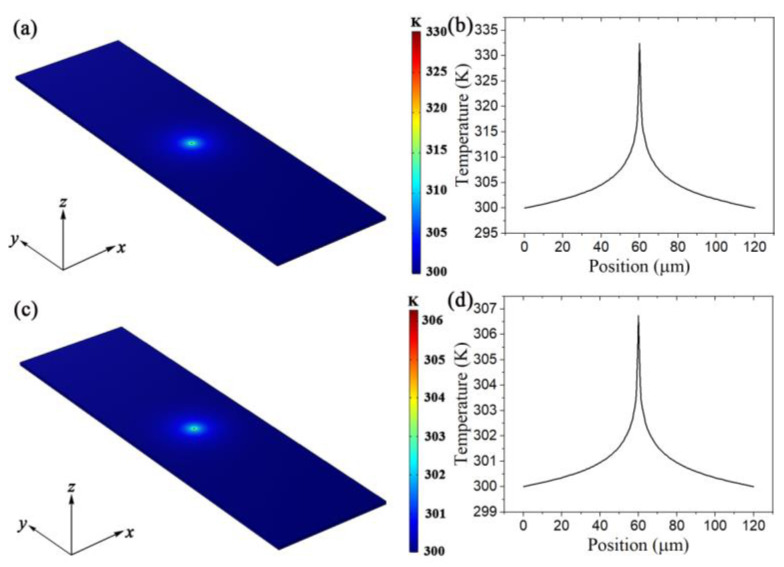
Simulated in-plane temperature distribution for (**a**) Sample 1, and (**c**) Sample 2. The temperature distributions of (**b**) Sample 1 and (**d**) Sample 2 along *x* axis at the center.

However, the calculation of an accurate solution to the temperature distribution *T*(*x*, *y*) at a steady state is very complicated. Here, we obtained the temperature distribution through numerical simulations with the following conditions: the length of the CNT film along the *x* axis was 120 μm; the width of the CNT film along the y axis was infinite; the thickness of the CNT film was 0.15 μm; the thermal conductivity of the CNT film was 6 W m^−1^ K^−1^ in the *xy*-plane and 0.1 W m^−1^ K^−1^ on the z axis; the density of the CNT film was 850 kg cm^−3^; the specific heat capacity of the CNT film was 1200 J kg^−1^ K^−1^; the heat transfer coefficient of air was 140 W m^−2^ K^−1^; the excitation laser power was 0.1 mW, the laser spot diameter was 0.5 μm; and the temperature of the air was maintained at a constant 300 K [37]. Because the length of the trench was much larger than the diameter of the laser spot, an assumption was introduced that the generated heat would completely transfer to the air before arriving at the substrate, and the influence of the substrate on the final temperature distribution could be neglected. This assumption proved to be true, as Figure S2 shows. The heat power transferred from the CNT film to the substrate was only 3% of the total heat dissipation, which demonstrated that the influence of the substrate was weak enough to be ignored. Figure 5a,c displays the simulated results for the temperature distribution. Figure 5b,d shows the temperature distributions of Sample 1 and Sample 2 along the x axis at the center. Considering that the temperature distribution under the illumination of the point light source indicates central symmetry, Equation (3) can be rewritten as:

(4) $$Q=Q_{surr}=2G_{0}\times \int_{0}^{l/2}(T-T_{0})2\pi r\cdot dr$$

where *l* is the length of the film along *x* axis. The PTCEs of Sample 1 (*η*_1_ = 0.26) and Sample 2 (*η*_2_ = 0.05) can be calculated by Equation (4). The average temperature within the laser light spot were consistent with the results calculated based on the value of *R*. Because the impurities in Sample 2 led to a reduced in-plane thermal conductivity, the real PTEC of Sample 2 should be smaller.

**Figure 6:**
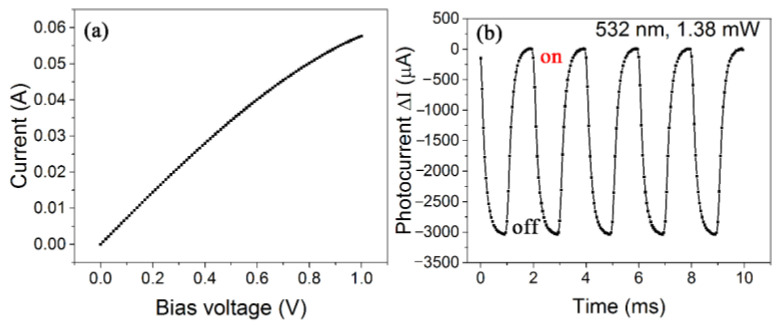
(**a**) *I–V* characteristic curve, whose linear range was extended to 0.8 V; (**b**) Photoresponse curve under 532 nm laser illumination in air; bias voltage was 0.8 V. The dark current was subtracted.

According to the above theoretical analyses and discussion, we tried to fabricate a bolometric photodetector based on a suspended CNT film with extremely high PTCE. We chose a thicker CNT film to reduce the defects caused by transfer, and then soaked the CNT film in a hydrogen peroxide solution and ethanol successively to remove the impurity (metal catalysts and amorphous carbon) in the films. Because the thickness of the CNT film was increased, the volt-ampere characteristic curve had a large linear range, as shown in Figure 6a. The photoresponse curve of the device under the illumination of a 532 nm laser is shown in Figure 6b. The device exhibited a responsivity of up to 2 A W^−1^, and the resistance change rate of the device was 4% mW^−1^, while the response time was only 0.2 ms. The performance of the device was much better than that of many photodetectors that only contained carbon nanotubes [15,16,25,37,38].

## 5. Conclusions

In conclusion, we proposed a simple, quick, and non-destructive method to measure the PTCE of different CNT films. Combining the increased temperature within the light spot and the simulated temperature distribution of the CNT films, the final values of the PTCE were calculated accurately. In addition, the impurities from metal catalysis and defects on the CNT film were demonstrated to have a great negative influence on the PTCE of the CNT film. Finally, a CNT film with the highest PTCE was identified by this method to be fabricated as a bolometric photodetector; the high performance (2 A W^−1^ at 532 nm) confirmed the effectiveness of the method. With the expanding applications of CNTs, this measurement method has huge potential in the future.

## Data Availability

The data presented in this study are available on request from the corresponding author.

## Supplementary Materials

The following supporting information can be downloaded at: https://www.mdpi.com/article/10.3390/nano12071101/s1, Figure S1: Characterization results of SEM for Sample 1 and Sample 2. Figure S2: Influence of the substrate on the temperature distribution.

## References

[1] Hu T., Chen K., Li L., Zhang J. (2021). Carbon nanotubes@silicone solar evaporators with controllable salt-tolerance for efficient water evaporation in a closed system. J. Mater. Chem. A.

[2] Jian H., Qi Q., Wang W., Yu D. (2021). A Janus porous carbon nanotubes/poly (vinyl alcohol) composite evaporator for efficient solar-driven interfacial water evaporation. Sep. Purif. Technol..

[3] Qin D.-D., Zhu Y.-J., Chen F.-F., Yang R.-L., Xiong Z.-C. (2019). Self-floating aerogel composed of carbon nanotubes and ultralong hydroxyapatite nanowires for highly efficient solar energy-assisted water purification. Carbon.

[4] Zhang Q., Xu W., Wang X. (2018). Carbon nanocomposites with high photothermal conversion efficiency. Sci. China Mater..

[5] Zhao Y., Yuan H., Zhang X., Xue G., Tang J., Chen Y., Zhang X., Zhou W., Liu H. (2021). Laser-assisted synthesis of cobalt@N-doped carbon nanotubes decorated channels and pillars of wafer-sized silicon as highly efficient three-dimensional solar evaporator. Chin. Chem. Lett..

[6] Zhu B., Kou H., Liu Z., Wang Z., Macharia D.K., Zhu M., Wu B., Liu X., Chen Z. (2019). Flexible and Washable CNT-Embedded PAN Nonwoven Fabrics for Solar-Enabled Evaporation and Desalination of Seawater. ACS Appl. Mater. Interfaces.

[7] Liang Hu S.G. (2015). Photothermal-Responsive Single-Walled Carbon Nanotube-Based Ultrathin Membranes for OnOff Switchable Separation of Oil-in-Water Nanoemulsions. ACS Nano.

[8] Chen Y., Zhang Q., Wen X., Yin H., Liu J. (2018). A novel CNT encapsulated phase change material with enhanced thermal conductivity and photo-thermal conversion performance. Sol. Energy Mater. Sol. Cells.

[9] Gao G., Zhang T., Guo C., Jiao S., Rao Z. (2020). Photo-thermal conversion and heat storage characteristics of multi-walled carbon nanotubes dispersed magnetic phase change microcapsules slurry. Int. J. Energy Res..

[10] Gimeno-Furió A., Martínez-Cuenca R., Mondragón R., Gasulla A.F.V., Doñate-Buendía C., Mínguez-Vega G., Hernández L. (2020). Optical characterisation and photothermal conversion efficiency of a water-based carbon nanofluid for direct solar absorption applications. Energy.

[11] Li B., Nie S., Hao Y., Liu T., Zhu J., Yan S. (2015). Stearic-acid/carbon-nanotube composites with tailored shape-stabilized phase transitions and light–heat conversion for thermal energy storage. Energy Convers. Manag..

[12] Behnam M.A., Emami F., Sobhani Z., Koohi-Hosseinabadi O., Dehghanian A.R., Zebarjad S.M., Moghim M.H., Oryan A. (2018). Novel Combination of Silver Nanoparticles and Carbon Nanotubes for Plasmonic Photo Thermal Therapy in Melanoma Cancer Model. Adv. Pharm Bull..

[13] Hashida Y., Tanaka H., Zhou S., Kawakami S., Yamashita F., Murakami T., Umeyama T., Imahori H., Hashida M. (2014). Photothermal ablation of tumor cells using a single-walled carbon nanotube-peptide composite. J. Control. Release.

[14] Murakami T., Nakatsuji H., Inada M., Matoba Y., Umeyama T., Tsujimoto M., Isoda S., Hashida M., Imahori H. (2012). Photodynamic and photothermal effects of semiconducting and metallic-enriched single-walled carbon nanotubes. J. Am. Chem. Soc..

[15] Liu Y., Hu Q., Yin J., Wang P., Wang Y., Wen J., Dong Z., Zhu J.-L., Wei J., Ma W. (2019). Bolometric terahertz detection based on suspended carbon nanotube fibers. Appl. Phys. Express.

[16] Liu Y., Yin J., Wang P., Hu Q., Wang Y., Xie Y., Zhao Z., Dong Z., Zhu J.L., Chu W. (2018). High-Performance, Ultra-Broadband, Ultraviolet to Terahertz Photodetectors Based on Suspended Carbon Nanotube Films. ACS Appl. Mater. Interfaces.

[17] He X., Fujimura N., Lloyd J.M., Erickson K.J., Talin A.A., Zhang Q., Gao W., Jiang Q., Kawano Y., Hauge R.H. (2014). Carbon nanotube terahertz detector. Nano. Lett..

[18] Zhang Y., Deng T., Li S., Sun J., Yin W., Fang Y., Liu Z. (2020). Highly sensitive ultraviolet photodetectors based on single wall carbon nanotube-graphene hybrid films. Appl. Surf. Sci..

[19] Harata T., Aono M., Kitazawa N., Watanabe Y. (2014). Correlation of photothermal conversion on the photo-induced deformation of amorphous carbon nitride films prepared by reactive sputtering. Appl. Phys. Lett..

[20] Tam N.T., van Trinh P., Anh N.N., Hong N.T., Hong P.N., Minh P.N., Thang B.H. (2018). Thermal Conductivity and Photothermal Conversion Performance of Ethylene Glycol-Based Nanofluids Containing Multiwalled Carbon Nanotubes. J. Nanomater..

[21] Zhou L., Wang X., Zhang J., Yang S., Hao K., Gao Y., Li D., Li Z. (2020). Self-suspended carbon nanotube/polyimide composite film with improved photothermal properties. J. Appl. Phys..

[22] Li Q., Liu C., Wang X., Fan S. (2009). Measuring the thermal conductivity of individual carbon nanotubes by the Raman shift method. Nanotechnology.

[23] Wu Q., Wen Z., Zhang X., Tian L., He M. (2018). Temperature Dependence of G− Mode in Raman Spectra of Metallic Single-Walled Carbon Nanotubes. J. Nanomater..

[24] Zhang Y., Xie L., Zhang J., Wu Z., Liu Z. (2007). Temperature Coefficients of Raman Frequency of Individual. J. Phys. Chem. C.

[25] Itkis M.E., Borondics F., Yu A., Haddon R.C. (2006). Bolometric infrared photoresponse of suspended single-walled carbon nanotube films. Science.

[26] Li Z., Jia Y., Wei J., Wang K., Shu Q., Gui X., Zhu H., Cao A., Wu D. (2010). Large area, highly transparent carbon nanotube spiderwebs for energy harvesting. J. Mater. Chem..

[27] Duzynska A., Taube A., Korona K.P., Judek J., Zdrojek M. (2015). Temperature-dependent thermal properties of single-walled carbon nanotube thin films. Appl. Phys. Lett..

[28] Liu Y., Hu Q., Wang P., Wei J., Huang F., Sun J.L. (2021). Electrically driven transport of photoinduced hot carriers in carbon nanotube fibers. Opt. Lett..

[29] Gao B., Jiang L., Ling X., Zhang J., Liu Z. (2008). Chirality-Dependent Raman Frequency Variation of Single-Walled Carbon Nanotubes under Uniaxial Strain. J. Phys. Chem. C.

[30] Liu M., Xiang R., Cao W., Zeng H., Su Y., Gui X., Wu T., Maruyama S., Tang Z. (2014). Is it possible to enhance Raman scattering of single-walled carbon nanotubes by metal particles during chemical vapor deposition?. Carbon.

[31] Shao Q., Que R., Shao M., Cheng L., Lee S.-T. (2012). Copper Nanoparticles Grafted on a Silicon Wafer and Their Excellent Surface-Enhanced Raman Scattering. Adv. Funct. Mater..

[32] Cao P.G., Yao J.L., Ren B., Mao B.W., Gu R.A., Tian Z.Q. (2000). Surface-enhanced Raman scattering from bare Fe electrode surfaces. Chem. Phys. Lett..

[33] Aghajani S., Accardo A., Tichem M. (2020). Aerosol Direct Writing and Thermal Tuning of Copper Nanoparticle Patterns as Surface-Enhanced Raman Scattering Sensors. ACS Appl. Nano Mater..

[34] García-Merino J.A., Martínez-González C.L., Miguel C.R.T.-S., Trejo-Valdez M., Martínez-Gutiérrez H., Torres-Torres C. (2015). Photothermal, photoconductive and nonlinear optical effects induced by nanosecond pulse irradiation in multi-wall carbon nanotubes. Mater. Sci. Eng. B.

[35] Zhang S., Mielke S.L., Khare R., Troya D., Ruoff R.S., Schatz G.C., Belytschko T. (2005). Mechanics of defects in carbon nanotubes: Atomistic and multiscale simulations. Phys. Rev. B.

[36] Li Z., Johnson O., Huang J., Feng T., Yang C., Liu Z., Chen W. (2018). Enhancing the photothermal conversion efficiency of graphene oxide by doping with NaYF4: Yb, Er upconverting luminescent nanocomposites. Mater. Res. Bull..

[37] Suzuki D., Kawano Y. (2020). Flexible terahertz imaging systems with single-walled carbon nanotube films. Carbon.

[38] St-Antoine B.C., Menard D., Martel R. (2009). Position Sensitive Photothermoelectric Effect in Suspended Single-Walled Carbon Nanotube Films. Nano Lett..

