# Supplementary material for: Measurement of the Photothermal Conversion Efficiency of CNT Films Utilizing a Raman Spectrum
Source: Nanomaterials (Basel). 2022 Mar 27;12(7):1101. doi: 10.3390/nano12071101 (PMC9000262; doi:10.3390/nano12071101)
Supplement: Supplementary file 1 [file nanomaterials-12-01101-s001.zip › nanomaterials-1630228-supplementary.pdf]

nanomaterials

Article

Measurement of the Photothermal Conversion Efficiency of CNT Films Utilizing a Raman Spectrum

Yu Liu [1], Zhicheng Lin [1], Pengfei Wang [1], Feng Huang [1] and Jia-Lin Sun [2,*]

[1] College of Mechanical Engineering and Automation, Fuzhou University, Fuzhou 350108, China; liuyu19@fzu.edu.cn (Y.L.); 15160454010@163.com (Z.L.); wangpf@fzu.edu.cn (P.W.); huangf@fzu.edu.cn (F.H.)

[2] State Key Laboratory of Low-Dimensional Quantum Physics, Department of Physics, Tsinghua University, Beijing 100084, China

* Correspondence: jlsun@mail.tsinghua.edu.cn

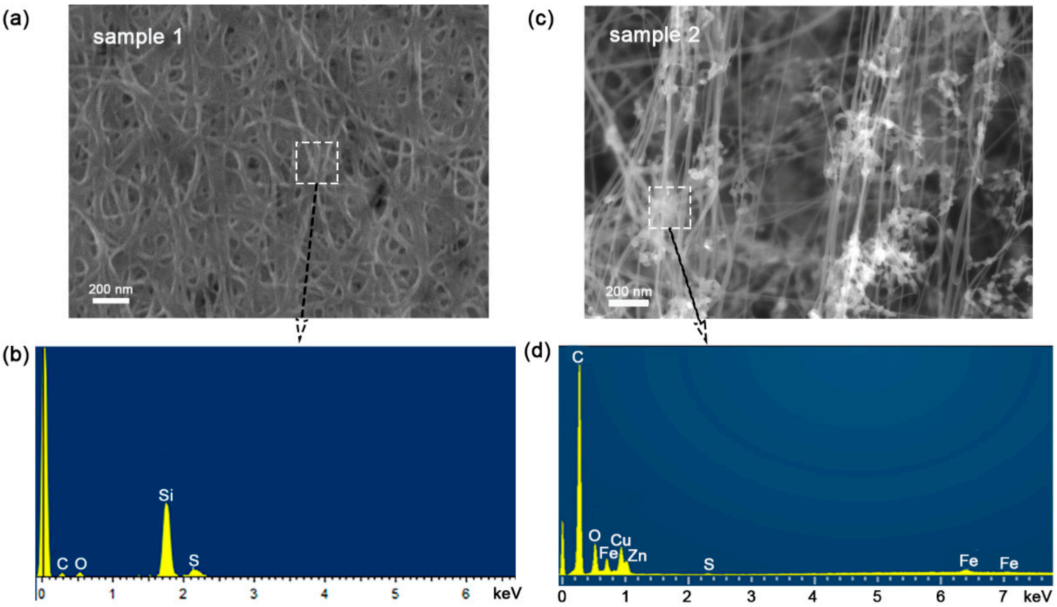

Figure S1. Characterization results of scanning electron microscope for (**a**) sample 1 and (**b**) sample 2. Results of elements analysis from x-ray energy dispersive spectroscopy for (**c**) sample 1 and (**d**) sample 2.

We simulated the temperature distribution of a larger CNT film which contacted with two substrates. The width of the contact area was 20 μm. The parameters of the Au film were same as Au block and the contact was intimate. Other parameters were same as those in main text. As Figure S2a,b shows the maximal temperatures of the two films are same. The temperature at the boundary of suspended film is almost equal to room temperature as Figure S2b shows.

To quantify the influence of the substrate, we enlarged the temperature distribution of the area contacted with substrate as Figure S2**c** shows. Only a very small area displayed a changed temperature. In addition, the temperature gradient along x direction at the boundary had also be calculated as Figure S2d shows. When the excitation light power was 0.1 mW, we found out the heat power transferred from CNT film to substrate was about 0.9 μW which was only 3% of the total heat dissipation. As a consequence, the influence of the substrate was demonstrated too weak, which can be ignored.

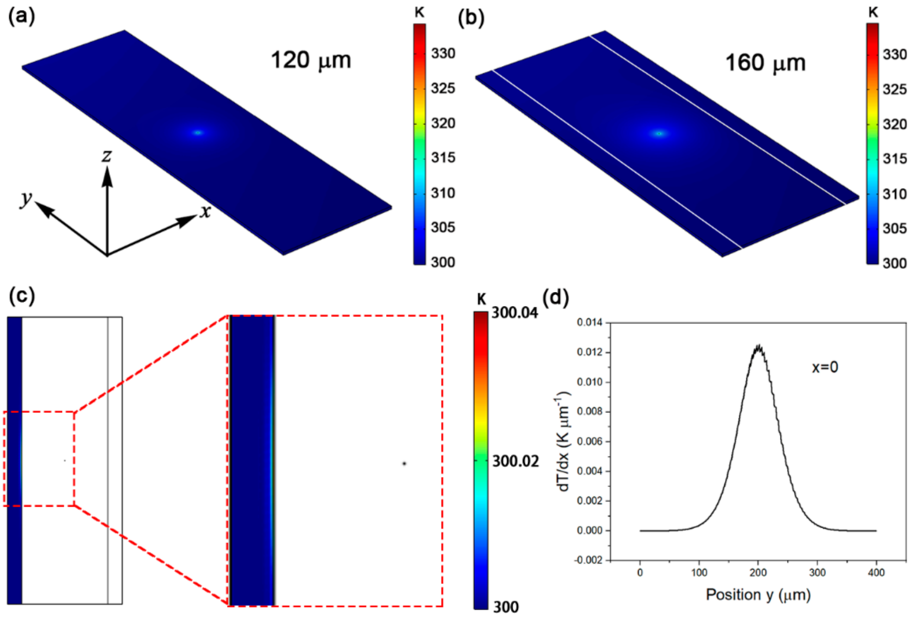

Figure S2. (**a**) Temperature of a CNT film neglecting the influence of substrate. (**b**) Temperature of a larger CNT film with two sides contacting with substrates. (**c**) Enlarged temperature distribution of the contact area. (**d**) Temperature gradient along the x direction at boundary.
